# Evaluation of the family medicine intern rotation in South Africa: A cross-sectional survey

**DOI:** 10.4102/safp.v68i1.6363

**Published:** 2026-10-15

**Authors:** Robert J. Mash, Febisola Ajudua, Mergan Naidoo, Klaus B. von Pressentin

**Affiliations:** 1 Division of Family Medicine and Primary Care, Faculty of Medicine and Health Sciences, Stellenbosch University, Cape Town, South Africa; 2 Department of Family Medicine and Rural Health, Faculty of Medicine and Health Sciences, Walter Sisulu University, Mthatha, South Africa; 3 Department of Family Medicine, Faculty of Health Sciences, Nelson Mandela University, Gqeberha, South Africa; 4 Department of Family Medicine, School of Medicine, College of Health Sciences, University of KwaZulu-Natal, Durban, South Africa; 5 Division of Family Medicine, Department of Family, Community and Emergency Care, University of Cape Town, Cape Town, South Africa

**Keywords:** interns, internship, training, district health services, primary care

## Abstract

**Background:**

South Africa introduced a 6-month rotation in family medicine (FM) as part of the 2-year internship. This necessitated a restructuring of the traditional hospital rotations. It is not known if the new rotation is meeting the goals set by the Health Professions Council of South Africa. The aim was to evaluate the new FM rotation for medical interns in South Africa.

**Methods:**

A descriptive cross-sectional survey of 260 interns, 74 supervisors across 34 intern programmes in all provinces of South Africa in 2025. Quantitative data were collected using a validated questionnaire, distributed using Redcap, and analysed descriptively in the Statistical Package for the Social Sciences.

**Results:**

Most interns ‘strongly agreed’ that they learnt how to refer patients (57.8%), how to manage people with chronic conditions (55.7%), and to take greater responsibility for clinical decision-making (53.9%). Most interns also ‘agreed’ that they learnt to coordinate care between the district and referral hospitals (72.8%), to consider the patient’s perspective (70.5%) and context (67.8%), to manage undifferentiated conditions (68.3%) and to rationalise prescribing (62.7%). Most interns reported little exposure to palliative care (58.2%), community-based services (56.0%) and quality improvement (50.2%). A substantial proportion reported little exposure to public health medicine (42.2%), continuity of care (26.2%) and forensic medicine (24.4%).

**Conclusion:**

The intern rotation in FM is fulfilling its promise in developing junior doctors who are fit for purpose and better able to cope with the community service year, with some specific areas for improvement.

**Contribution:**

The findings confirm the value of the rotation and should guide quality improvement.

## Introduction

In 2021, the Health Professions Council of South Africa (HPCSA) introduced a 6-month family medicine (FM) rotation during the second year of internship training.^1^ The increased duration was aimed at capacitating interns for work in the district health services of South Africa.^2^ The goals set for the training include specific objectives in improving management of undifferentiated conditions in primary care, disease surveillance, increased awareness of district health services and clinical governance.^1^ The end-point is that medical doctors, on completion of the internship training in South Africa, are able to work in district hospitals and primary care facilities during their community service in line with the requirements of the HPCSA.^1^

The previous 2-year medical internship programme in South Africa expected the intern to rotate through six domains of 4 months each.^1^ The FM domain included 3 months of FM and 1 month of psychiatry, and could be completed in either the first or the second year. In 2021, a new 2-year programme consisted of four domains (3 months of paediatrics, surgery, internal medicine and obstetrics & gynaecology) in the first year and a second year that consisted of three domains (2 months of anaesthetics, orthopaedics and psychiatry) and a 6 month rotation in FM.^3^ Exposure to FM has therefore increased from 3 months in either first or second year to 6 months in the second year.

Across the country, internship training has traditionally taken place in accredited tertiary or regional hospitals.^1^ The initial introduction of a rotation in FM required these hospitals to incorporate district hospitals and primary care facilities into their training complexes. It also promoted the inclusion of family physicians as trainers and intern curators. Supervision, however, does not have to be from a family physician and can be provided by any medical officer with more than 3 years of post-internship experience. The more recent increase in time committed to FM and primary care has necessitated a reduction in time spent in the traditional major disciplines (paediatrics, surgery, internal medicine and obstetrics & gynaecology). Inevitably, this has raised questions about the value of increased exposure to FM and primary care.^4^

The aim of this study was to evaluate the new FM rotation for medical interns at district health facilities in all nine provinces of South Africa. Specific objectives were to:

- Describe the different exposures to learning opportunities and settings.
- Evaluate the learning of medical interns and how they developed as health professionals.
- Identify the strengths and weaknesses of the rotation.

## Research methods and design

### Study design

This was a cross-sectional descriptive survey of interns and their supervisors in the FM internship rotation.

### Setting

The FM medical internship is a 6-month rotation in the second year of the medical internship. A training programme is usually based at a secondary or tertiary level hospital where interns rotate for 2 years through the designated domains. In FM, interns are meant to work in primary care facilities, district hospitals and emergency centres. The interns are supervised by family physicians and medical officers. Each discipline will have a senior doctor to coordinate their rotation and each training programme an intern curator to ensure the seamless transition of medical interns from one clinical rotation to another.

### Study population, sample size and sampling

This study intended to evaluate the intern programme in all provinces. All second-year interns and their supervisors participating in the 6-month FM rotation in any of the 66 training programmes in South Africa were included in the study population. Asynchronous medical interns who had not completed 5 months of rotation in FM were excluded.

There were an estimated 2473 interns across 66 intern training programmes in South Africa rotating through FM, according to the HPCSA list of accredited facilities. This was half of the total number of 4946, as only second-year interns rotate to FM. A sample size calculation was based on this population size, with 95% confidence intervals, a 5% margin of error and a response proportion of 50%, yielding a sample size of 333.^5^ This was increased to a target of 350 to allow for some loss of data.

Stratified random sampling was used to attain the required sample size. First of all, the sample was stratified by province according to the proportion of all interns in each province (Table 1). The number of interns required from each province was then divided by 10 to ensure a feasible number of programmes were included in the survey. The required number of programmes was then selected at random from the total list of programmes in each province. The number of interns required from each of these selected programmes was then stratified by the proportion of interns in each programme. The final sample consisted of 36 programmes and 350 interns.

**TABLE 1:** Overview of stratified sampling.

Province	Number of interns	%	Sample size	Number of programmes
Gauteng	796	32.2	113	11
KwaZulu-Natal	532	21.5	75	8
Western Cape	339	13.7	48	5
Eastern Cape	216	8.7	31	3
North West	172	7.0	24	2
Limpopo	170	6.9	24	2
Free State	108	4.4	15	2
Mpumalanga	100	4.0	14	2
Northern Cape	40	1.6	6	1

**Total**	**2473**	**100.0**	**350**	**36**

It was difficult to determine the exact number of supervisors responsible for training at these sites, but all of the supervisors at these sites were invited to complete the survey. There is no register of the number of supervisors in each programme. Supervisors are those directly responsible for the training at different training sites (e.g. primary care facility, district hospital) within the intern programme and the person overall responsible for the rotation. We invited a minimum of two respondents per selected programme, giving a minimum sample size of 72 supervisors. We intended a response rate of at least 80%.

### Data collection

The questionnaires for interns and supervisors were already designed and validated as part of a sequential exploratory mixed methods study in the Western Cape.^6,7^ The questionnaire was further validated by the research committee of the South Africa Academy of Family Physicians. This committee consisted of FM researchers across South Africa, and they ensured the content was generally applicable to other provinces. The questionnaire was in English. The final intern questionnaire had four sections. Section A collected demographic information, Section B presented respondents with statements about their learning and training experience using a four-point Likert scale (strongly disagree, disagree, agree, strongly agree), Section C asked them to rank aspects of the rotation from most to least important, while Section D asked them to rank the type of supervisor from most to least important. The supervisor questionnaire had the same structure for Sections A and B, but omitted Sections C and D that could only be answered by the interns.

Data were collected at the end of each rotation (June – July 2025 and December 2025 – January 2026). The questionnaires were created in REDCap, an electronic survey platform accessed through a link sent to invited participants. The family medicine coordinator in each programme disseminated the link to randomly selected interns, based on the required sample size. They completed the questionnaire themselves and invited at least one other supervisor. Reminders were sent until the sample size was obtained or no further response was likely in January 2026.

### Data analysis

Data were exported from REDCap into the Statistical Package for the Social Sciences (IBM^®^ SPSS^®^ Statistics 30.0) and analysed by the principal researcher. Categorical data is described as frequencies and percentages, while numerical data is described as means and standard deviations or medians and interquartile ranges (IQRs), depending on its distribution.

### Ethical considerations

Ethical clearance to conduct this study was obtained from the Stellenbosch University Health Research Ethics Committee. The ethical clearance number is N25/03/024. Also, separate permissions from the Department of Health in each province were received to conduct the study (WC/ NC/ EC/ MP/ GP/ LP/ FS/ KZN/ NW_202505_028).

## Results

There were 260 (74% response rate) completed questionnaires from interns, 174 (66.9%) were women and 86 (33.1%) were men, with a median age of 26 years (IQR 25–28). Ninety-three interns (35.8%) were from the first rotation of 2025, 151 (58.1%) from the second rotation and 16 (6.2%) from the first rotation of 2026.

Overall, 74 supervisors completed their questionnaire, 36 (48.6%) were women and 38 (51.4%) were men, with a median age of 48 years (IQR 40–57); 61 (82.4%) were clinical trainers and 13 (17.6%) coordinators of the programme. Table 2 shows the distribution of intern and supervisor responses between provinces. The results of the intern and supervisor surveys are integrated below to give a more complete picture of the intern rotation.

**TABLE 2:** Characteristics of the respondents.

Province	Interns (*N* = 260)		Supervisors (*N* = 73)	
	*n*	%	*n*	%
Gauteng	79	30.4	19	26.0
KwaZulu-Natal	66	25.4	16	21.9
Western Cape	37	14.2	20	27.4
Eastern Cape	21	8.1	4	5.5
Free State	16	6.2	3	4.1
Northern Cape	14	5.4	3	4.1
Limpopo	13	5.0	3	4.1
Mpumalanga	8	3.1	2	2.7
North West	6	2.3	3	4.1

The supervisors provided data from 33 intern programmes, and the exposure to different types of facilities over 24 weeks is shown in Table 3. All programmes included exposure to primary care facilities and district hospitals, and most also included the emergency centre in the regional or base hospital. Placement at the emergency centre exceeded 10 weeks in 2 programmes, whereas placement at the primary care facility lasted only 2–4 weeks in 12 programmes. Other types of exposure were offered by 12 programmes, and these included public health, specialised clinics (e.g. HIV, diabetes), old age homes or hospice. At the district hospital, interns reported exposure to outpatients (*n* = 181, 69.6%), adult wards (*n* = 171, 65.8%), maternity (*n* = 140, 53.8%), the operating theatre (*n* = 131, 50.4%) and the children’s ward (*n* = 114, 43.8%). Only 58 (22.3%) reported exposure to community health workers, and 17 (6.5%) to hospice or transitional care.

**TABLE 3:** Structure of the intern rotations between different types of facilities (*N* = 33).

Weeks of exposure	Emergency centre in the base hospital (*n*)	District hospital (*n*)	Primary care facility (*n*)	Other (*n*)
0	4	0	0	21
2–4	15	7	12	9
6–8	12	13	16	3
10–12	1	8	3	0
14–16	1	3	2	0
18–20	0	1	0	0
22–24	0	1	0	0

In Table 4, the interns give feedback on their learning during the rotation. The majority of interns achieved key learning outcomes in providing chronic care (96.5%), managing undifferentiated problems (96.1%), managing new conditions they had not encountered in earlier rotations (96.9%), rational prescribing (90.7%), performing new procedures (81.8%) and consolidating other procedures (87.2%), managing mental health disorders (85.9%) and adopting a more holistic approach (the person 94.7% and their context 92.5%). They also learnt about referral pathways (98.2%), although coordinating care with community health workers was a gap for many interns (43.8%). Overall, they grew in confidence (99.5%) and resilience (97.8%) and took more responsibility for patient care than in previous rotations (96.9%). The supervisors (61, 93.8%) agreed that the rotation enabled substantial personal and clinical growth.

**TABLE 4:** Interns’ feedback on their learning during the family medicine rotation (*N* = 260).

Outcome	Strongly disagree and disagree		Agree		Strongly agree	
	*n*	%	*n*	%	*n*	%
I learnt how to refer a patient from the district health services to regional or tertiary hospitals (*N* = 225)	4	1.8	91	40.4	130	57.8
I learnt how to provide ongoing care for people with chronic conditions (*N* = 228)	8	3.5	93	40.8	127	55.7
I took more responsibility for patient care and clinical decision-making in this rotation (*N* = 228)	7	3.1	98	43.0	123	53.9
I gained confidence in dealing with common presenting problems in primary care (*N* = 225)	1	0.4	113	50.2	111	49.3
I learnt how to develop my own resilience and cope with the workload (*N* = 225)	5	2.2	128	56.9	92	40.9
I was able to gain more experience in certain procedures (*N* = 227)	29	12.8	106	46.7	92	40.5
I learnt how to manage a variety of new acute conditions (*N* = 226)	7	3.1	130	57.5	89	39.4
I learnt how to perform a number of new procedures (*N* = 226)	41	18.1	121	53.5	64	28.3
I learnt about rational prescribing in the district health setting (*N* = 225)	21	9.3	141	62.7	63	28.0
I learnt how to assess patients who presented with an undifferentiated problem (*N* = 227)	9	4.0	155	68.3	63	27.8
I learnt about managing people with mental health problems (*N* = 227)	32	14.1	132	58.1	63	27.8
I learnt to consider the family and community context more when assessing patients (*N* = 227)	17	7.5	154	67.8	56	24.7
I learnt to consider why the patient had come for help now and their perspective on the illness (*N* = 227)	12	5.3	160	70.5	55	24.2
I learnt how to coordinate care between primary care and the district hospital (*N* = 224)	18	8.0	163	72.8	43	19.2
I learnt how to coordinate care with community health worker teams in the community (*N* = 224)	98	43.8	100	44.6	26	11.6

Table 5 summarises the interns’ feedback on their educational exposure to various activities during the rotation. Although the majority agreed that they participated in activities to improve quality care and patient safety (84.5%), only 49.8% actually participated in a quality improvement project. Although the majority reported exposure to continuity of care, forensic medicine and public health, a substantial proportion did not have this experience (26.2%, 24.4% and 42.2%, respectively). The majority did not have any exposure to palliative care (58.2%) or community-based services (56.0%).

**TABLE 5:** Interns’ feedback on their educational exposure to different activities.

Educational activity	Strongly disagree and disagree		Agree		Strongly agree	
	*n*	%	*n*	%	*n*	%
I was able to participate in activities to improve the quality of care and patient safety (*N* = 225)	35	15.6	139	61.8	51	22.7
I was able to see the same patient over time and have some continuity of care (*N* = 225)	59	26.2	126	56.0	40	17.8
I was able to participate in a quality improvement project (*N* = 225)	113	50.2	76	33.8	36	16.0
I was able to participate in forensic health services (*N* = 225)	55	24.4	139	61.8	31	13.8
I was able to engage with public health medicine (*N* = 225)	95	42.2	101	44.9	29	12.9
I was able to provide care in the community (*N* = 225)	126	56.0	73	32.4	26	11.6
I was able to participate in palliative care services (*N* = 225)	131	58.2	87	38.7	7	3.1
There was too much focus on emergency care and not enough exposure to chronic conditions (*N* = 227)	199	87.7	24	10.6	4	1.8

In Table 6, interns give feedback on the more formal teaching and educational guidance that was available. The majority reported a supportive culture of learning and a structured teaching programme at the base hospital. A substantial proportion reported no such teaching at district hospitals (29.8%) or in primary care (44.0%), and no access to a continuing professional development (CPD) programme for staff (35.4%). The majority of supervisors reported (55, 84.6%) that structured teaching was available. Most interns reported that the HPCSA logbook guided learning, helped assess progress and was well aligned with actual learning opportunities. However, many supervisors (29, 44.6%) reported that the logbook was not completed timeously throughout the rotation. Most interns (60%) reported no access to a mentorship programme, although only 44.6% of supervisors agreed with this feedback.

**TABLE 6:** Intern feedback on formal teaching and educational guidance.

Items	Strongly disagree and disagree		Agree		Strongly agree	
	*n*	%	*n*	%	*n*	%
There was a structured programme of teaching for interns at the base hospital (*N* = 225)	40	17.8	135	60.0	50	22.2
The facilities where I worked had an established and supportive culture of learning (*N* = 225)	37	16.4	141	62.7	47	20.9
The HPCSA logbook guided me in what I should be learning during the rotation (*N* = 225)	30	13.3	150	66.7	45	20.0
The HPCSA logbook helped me assess my progress during the rotation (*N* = 225)	37	16.4	147	65.3	41	18.2
What I actually learnt was well aligned with the outcomes written in the HPCSA logbook (*N* = 225)	24	10.7	164	72.9	37	16.4
There was a structured programme of teaching for the interns at the district hospital (*N* = 225)	67	29.8	125	55.6	33	14.7
I was able to join the continuing professional development programme with other staff (*N* = 223)	79	35.4	113	50.7	31	13.9
There was a structured programme of teaching for interns at the primary care facility (*N* = 225)	99	44.0	101	44.9	25	11.1
A mentorship programme for junior doctors was present at my facility (*N* = 225)	135	60.0	75	33.3	15	6.7

HPCSA, Health Professions Council of South Africa.

Table 7 reports on supervision from the perspective of both the interns and supervisors. The majority of the interns found the supervisors and coordinators approachable and helpful. Most were also satisfied with the amount of supervision during office hours and after hours. A substantial number (32.0%) reported inadequate supervision in the primary care facility. Most supervisors reported adequate supervision, although primary care was the weakest area. A substantial number of supervisors (33.9%) reported that providing supervision was a challenge, and that ensuring supervision was not easy (41.5%).

**TABLE 7:** Feedback on supervision.

Items	Strongly disagree and disagree		Agree		Strongly agree	
	*n*	%	*n*	%	*n*	%
**Intern results (*N* = 225)**						
The intern supervisor(s) were approachable and helpful during the rotation	13	5.8	129	57.3	83	36.9
The intern curator was approachable and helpful during the rotation	28	12.4	109	48.4	88	39.1
I had adequate supervision during the day, which enabled me to learn without risking harm to patients or myself	31	13.8	114	50.7	80	35.6
I had adequate supervision after hours, which enabled me to learn without risking harm to patients or myself	36	16.0	122	54.2	67	29.8
There was too much supervision; I was never left to make my own decisions	207	92.0	13	5.8	5	2.2
I was left alone at the primary healthcare level with inadequate supervision	153	68.0	55	24.4	17	7.6
**Supervisor results (*N* = 65)**						
Providing supervision of interns was an easy task	29	41.5	28	43.1	8	12.3
It was a challenge to always provide supervision for interns	43	66.2	20	30.8	2	3.1
Interns were always supervised during daytime hours at the district hospital	9	13.8	31	47.7	25	38.5
Interns were always supervised during daytime hours in primary care	12	18.5	28	43.1	25	38.5
Interns were always supervised during daytime hours in the emergency centre	6	9.2	21	32.3	38	58.5
Interns were always supervised during after-hours activities	5	7.7	33	50.8	27	41.5

Interns ranked who they received the most clinical support and supervision from. From most to least important, they listed medical officers, community service medical officers, nurse practitioners, family physicians, FM registrars, other specialists and lastly, allied health practitioners.

Table 8 reports on the experience of service delivery from both the perspective of interns and supervisors, as well as the overall assessment of the rotation. Both interns and supervisors agreed that they made a significant contribution to service delivery, were well integrated into clinical teams, and were not a clinical or administrative burden. They were able to apply lessons learnt from prior rotations and assume greater responsibility for clinical decision-making. Interns ranked aspects of the rotation (from highest to lowest) and identified clinical confidence, managing undifferentiated problems, personal growth, clinical procedures, managing complex patients and working in multidisciplinary teams as the top six options.

**TABLE 8:** Feedback on service delivery, teamwork and rotation.

Items	Strongly disagree and disagree		Agree		Strongly agree	
	*n*	%	*n*	%	*n*	%
**Intern results**						
I recognised the value of family medicine as a speciality (*N* = 225)	13	5.8	117	52.0	95	42.2
My intern colleagues and I made a clear difference to health services (*N* = 225)	5	2.2	127	56.4	93	41.3
The family medicine rotation is the best rotation in preparation for the community service year (*N* = 224)	21	9.4	113	50.4	90	40.2
During this rotation, I was able to put into practice all that I have learnt in the other rotations (*N* = 225)	7	3.1	129	57.3	89	39.6
I was acknowledged and included as a full member of the clinical team (*N* = 225)	20	8.9	130	57.8	75	33.3
My clinical assessments and opinions were trusted by my senior colleagues (*N* = 225)	3	1.3	151	67.1	71	31.6
The district hospital/primary care environment is more conducive to fostering independence in clinical decision-making compared to the specialist hospital environment (*N* = 223)	16	7.2	137	61.4	70	31.4
At the beginning of the rotation, I was orientated through a structured programme to understand what would be expected from me (*N* = 225)	33	14.7	125	55.6	67	29.8
I felt more connected with the clinical team in the district hospital/primary care environment compared to the specialist hospital environment (*N* = 224)	65	29.0	100	44.6	59	26.3
The family medicine rotation was my favourite rotation during my internship (*N* = 225)	124	55.1	71	31.6	30	13.3
I was just an extra pair of hands, used to fill gaps (*N* = 225)	153	68.0	49	21.8	23	10.2
**Supervisor**						
Interns were valuable members of the clinical team (*N* = 65)	2	3.1	16	24.6	47	72.3
Interns were seen as a burden by other medical officers (*N* = 65)	59	90.8	4	6.2	2	3.1
Interns added extra admin for managers and supervisors (*N* = 65)	45	69.2	16	24.6	4	6.2
The interns had difficulty making clinical decisions, which negatively impacted service delivery (*N* = 65)	53	81.5	10	15.4	2	3.1
Interns were inexperienced and needed a lot of extra help when on duty (*N* = 65)	39	60.0	25	38.5	1	1.5
The interns were supernumerary and did not fit well with the team (*N* = 65)	63	96.9	2	3.1	0	0.0

The majority felt that the rotation helped them recognise the value of FM (94.2%) and that it best prepared them for community service (90.6%). A substantial proportion (44.9%) said this was their favourite rotation. The majority of interns (179/224, 79.9%) and supervisors (54/65, 83.1%) thought the 6-month duration was ‘just right’. Some interns thought it was too long (37, 16.5%), and some supervisors thought it was too short (8/65, 12.3%).

## Discussion

### Summary of key findings

Interns achieved most of the key HPCSA learning outcomes, and almost all saw this as the best rotation to prepare them for community service and agreed with the 6-month duration. Interns ranked clinical confidence, managing undifferentiated problems, personal growth, clinical procedures, managing complex patients and working in multidisciplinary teams as the key areas of learning. However, some aspects of the rotation could be improved. Interns were not exposed to community-based services, and specifically, community health workers. Exposure to public health was likewise poor in many programmes, and less than half were involved in quality improvement work. Exposure to palliative care and forensic medicine could also be improved. Despite working in primary care, their experience of continuity of care was limited.

Formal teaching opportunities diminished as you moved away from the base hospital into primary care. Many could not participate in the local CPD programmes, and most had no access to mentorship. Support and supervision came primarily from medical officers, including community service medical officers. Nurse practitioners provided more support and supervision than family physicians and registrars. Overall supervision was adequate after hours and during the day, but was least available in primary care.

### Discussion of key findings

Most rotations had district hospital exposure and primary care as the foundation, although a few had too much emphasis on work in the regional hospital emergency centre and too little time in primary care. A previous report from Mpumalanga concluded that 2 months at the district hospital and primary care platform, compared with the base hospital, were not sufficient.^8^ In the district hospital, interns were less exposed to working in the children’s ward. Overall, interns were regarded as valuable members of healthcare teams and were not experienced as an additional burden.

Overall, the rotation is delivering on its ‘exceptional potential in extending primary care exposure for South African medical interns’.^9^ The district health services expose interns to undifferentiated problems at an earlier stage of their natural history. Patients present with significant medical issues, but also with problems of living, concerns about symptoms and requests for preventative interventions.^10^ Patients also present with diseases that are seldom seen in referral hospitals but may be common in communities. Feedback suggested that interns were incorporated into multidisciplinary teams and given more responsibility for decision-making, which helped to build clinical confidence and personal growth. Care for diseases such as HIV, TB, diabetes, hypertension, ischaemic heart disease, asthma and chronic obstructive pulmonary disease mainly takes place in primary care,^11^ and the rotation developed skills in ongoing chronic care. Primary care also gave interns the opportunity to learn from and support nurse practitioners, thereby helping them prepare for interprofessional collaborative practice.^12^

The strengths and weaknesses of the rotation mostly reflect the same issues seen in district health services. Primary healthcare re-engineering introduced ward-based outreach teams and community health workers,^13^ but in many areas, teams struggle with supportive supervision, integration with the facility-based multidisciplinary teams and sufficient resources.^14^ Although some provinces are promoting community-oriented primary care approaches, implementation at scale is ongoing. There needs to be a shift from a focus on individual patients in the facility to the community as a whole. Although coronavirus disease 2019 (COVID-19) highlighted the value of an integrated primary care and public health approach,^15^ this lesson may not have been sustained post-COVID-19. It is therefore not surprising that interns were retained within facilities and had minimal exposure to community-based services.

Palliative care has been gaining recognition in South Africa.^16^ A sub-speciality of palliative care is being promulgated, and FM will be one of the foundational disciplines. National policy supports the implementation of palliative care in district health services.^17^ However, in many provinces, the implementation and integration of palliative care is aspirational. Interns, therefore, might struggle to include palliative care in their learning opportunities.

Interns were exposed to many of the core functions of primary care.^18^ They learnt how to coordinate care and understood the issues with referring patients to the hospital. They appreciated the need for comprehensive care, which often involves multimorbidity and complexity. Interns reported that they had to adopt a more holistic, and therefore more person-centred, approach to patients. Continuity of care, however, was an issue, and this has been recognised in evaluations of primary care performance.^19^ Our primary care services struggle with informational continuity in the absence of an electronic medical record and with relational continuity as patients typically see a different primary care provider at each visit.

Provinces only employ 132 family physicians in district hospitals and community health centres.^20^ The distribution is also skewed, with 55% of eligible facilities in Gauteng having a family physician and 0% in the Northern Cape. On the one hand, the intern programme is increasing awareness and interest in FM as a career, but on the other hand, provinces are not employing sufficient family physicians to create career pathways. One consequence of this is a limited supply of family physicians to supervise and train interns.

### Strengths and limitations

The study achieved 78% (260/333) of the required sample size for the interns, despite two programmes in Gauteng that did not participate. Multiple attempts were made to obtain the sample size, but once interns had left for community service, it was difficult to contact them. Intern curators and supervisors also varied in their motivation to assist with data collection. Despite this, the distribution of interns in the sample mirrors that in the country and is likely representative. A few interns from the first rotation in 2026 also completed the questionnaire. They had not completed the rotation and may have had a more limited experience. It is unlikely that their responses would change the key findings.

The minimum expected sample size for the supervisors was attained with a good distribution across the provinces, although the proportion from the Western Cape was higher than expected. The sample was not powered to compare provinces or programmes, and so inferential analysis was not performed.

### Recommendations

The 6-month FM internship should be retained as a core component of the internship. Interns are a significant asset to service delivery, and districts should not hesitate to include interns in their services. Intern curators and FM supervisors can strengthen the rotation by ensuring adequate exposure to primary care, including community-based services. Exposure to palliative care, patients with forensic issues, public health functions and clinical governance activities should also be included.

Supervision is not meant to rely on community service doctors or nurse practitioners, although these are often the most readily available sources of support, along with medical officers. Family physicians and FM registrars should explore ways to be more available. Registrars need to develop competencies in clinical training, and engagement with interns also fulfils their learning needs. Provinces should also employ more family physicians, in this case, to enable proper supervision of interns.

Ideally, interns should receive more formal clinical teaching. This is a struggle in many facilities with high workloads and other training commitments. However, an internship is meant to be more of an apprenticeship with on-the-job clinical training, and they should be able to access the continuing professional development programmes offered to all facility-level staff. Programmes may want to consider the use of digital technology, which could provide online teaching to larger groups of interns and spread the load across multiple teachers. This could even be organised by a national body such as the South African Academy of Family Physicians.

## Conclusion

Interns gained valuable competencies in managing undifferentiated conditions and chronic care. Their clinical confidence increased, and they experienced significant personal growth. They encountered new conditions and learnt new procedures, while consolidating learning from prior rotations. Rotations all combined exposure to district hospitals and primary care, although some had too much exposure to the base hospital emergency centre and too little in primary care. Interns found this rotation to be the best way to prepare them for community service and made valuable contributions to health services.

The rotation could be strengthened by more exposure to community-based services, palliative and forensic care, public health and clinical governance. The supply of family physician supervisors was limited, and many interns relied on community service doctors and nurse practitioners for support. Interns should have access to continuing professional development programmes, and consideration should be given to bespoke online teaching as an efficient educational method.
